# Catheter ablation in patients with paroxysmal atrial fibrillation and absence of structural heart disease: A meta-analysis of randomized trials

**DOI:** 10.1016/j.ijcha.2023.101292

**Published:** 2023-11-05

**Authors:** Antonio Parlavecchio, Giampaolo Vetta, Giovanni Coluccia, Lorenzo Pistelli, Rodolfo Caminiti, Manuela Ajello, Michele Magnocavallo, Giuseppe Dattilo, Rosario Foti, Scipione Carerj, Pasquale Crea, Michele Accogli, Gian Battista Chierchia, Carlo de Asmundis, Domenico Giovanni Della Rocca, Pietro Palmisano

**Affiliations:** a Cardiology Unit, Department of Clinical and Experimental Medicine, University of Messina, Messina, Italy; b Cardiology Unit, “Card. G. Panico” Hospital, Via S. Pio X, 73039 Tricase, Italy; c Arrhythmology Unit, Ospedale San Giovanni Calibita, Fatebenefratelli Isola Tiberina, Via Ponte Quattro Capi 39,00186 Rome, Italy; d San Vincenzo Hospital, Taormina, Italy; e Heart Rhythm Management Centre, Postgraduate Program in Cardiac Electrophysiology, and Pacing, Universitair Ziekenhuis Brussel-Vrije Universiteit Brussel, European, Reference Networks Guard-Heart, Brussels, Belgium

**Keywords:** Atrial fibrillation, Radiofrequency, Pulmonary vein isolation, Cryoablation, Antiarrhythmic drugs

## Abstract

**Introduction:**

Rhythm control strategy in paroxysmal atrial fibrillation (AF) can be performed with antiarrhythmic drugs (AAD) or catheter ablation (CA). Nevertheless, a clear overview of the percentage of freedom from AF over time and complications is lacking. Therefore, we conducted a *meta*-analysis of randomized controlled trials (RCTs) comparing CA versus AAD.

**Methods:**

We searched databases up to 5 May 2023 for RCTs focusing on CA versus AAD. The study endpoints were atrial tachyarrhythmia (AT) recurrence, progression to persistent AF, overall complications, stroke/TIA, bleedings, heart failure (HF) hospitalization and all-cause mortality.

**Results:**

Twelve RCTs enrolling 2393 patients were included. CA showed a significantly lower AT recurrence rate at one year [27.4 % vs 56.3 %; RR: 0.45; p < 0.00001], at two years [39.9 % vs 62.7 %; RR: 0.56; p = 0.0004] and at three years [45.7 % vs 80.9 %; RR: 0.54; p < 0.0001] compared to AAD. Furthermore, CA significantly reduced the progression to persistent AF [1.6 % vs 12.9 %; RR: 0.14; p < 0.00001] with no differences in overall complications [5.9 % vs 4.5 %; RR: 1.27; p = 0.22], stroke/TIA [0.6 % vs 0.6 %; RR: 1.10; p = 0.86], bleedings [0.4 % vs 0.6 %; RR: 0.90; p = 0.84], HF hospitalization [0,3% vs 0,7%; RR: 0.56; p = 0.37] and all-cause mortality [0,4% vs 0.5 %; RR: 0.78; p = 0.67]. Subgroup analysis between radiofrequency and cryo-ablation or considering RCTs with CA as first-line treatment showed no significant differences.

**Conclusion:**

CA demonstrated lower rates of AT recurrence over the time, as well as a significant reduction in the progression from paroxysmal to persistent AF, with no difference in terms of energy source, complications, and clinical outcomes.

## 1 Introduction

Atrial fibrillation (AF) is the most common sustained cardiac arrhythmia, affecting a significant proportion of the global population. It is associated with an increased risk of stroke, bleeding, heart failure (HF), and mortality [1], [2], [3], [4]. EAST-AFNET 4 demonstrated that early rhythm control strategy is associated with a lower risk of adverse cardiovascular outcomes compared to usual care among patients with AF [5].

Rhythm control strategy in AF can be performed with antiarrhythmic drugs (AAD) or catheter ablation (CA).

In recent years, several randomized trials compared the efficacy and safety of CA and AAD in patients with paroxysmal AF without structural heart disease (SHD) [6], [7], [8], [9], [10], [11], [12], [13], [14], [15], [16], [17].

Trials comparing the two strategies showed reduced AF recurrence and less progression from paroxysmal to persistent AF with CA [6], [7], [9], [13]. However, a clear overview of long-term freedom from AF recurrences is lacking. Furthermore, no difference in terms of complications and clinical outcomes was observed between the two groups in patients with paroxysmal AF without SHD [6], [11], [14], [16], [18].

Therefore, we conducted a *meta*-analysis of randomized trials with the aim of comparing freedom from AF, progression to persistent AF, overall complication rate and clinical outcomes between CA and AAD.

## 2 Methods

### 2.1 Data sources and searches

We systematically searched the Medline, Embase and Scopus electronic databases for studies published from the time of inception to May 5th 2023 and focusing on CA versus AAD in paroxysmal AF patients. Two investigators (A.P. and G.V.) independently performed searches including the following terms: “ablation and drug therapy paroxysmal atrial fibrillation”. Detailed information of our literature search strategy is available in Supplemental Material in the Expanded Methods. The study protocol was designed before the start of the literature search but was not registered in any database.

### 2.2 Study selection

The Preferred Reporting Items for Systematic reviews and Meta-Analyses (PRISMA) statement for reporting systematic reviews and *meta*-analyses was used in this study [19].

Only RCTs were included to reduce the intrinsic bias due to the nature of non-randomised observational studies.

The studies had to fulfil the following criteria to be included in the analysis: (1) presence of a direct comparison between CA and AAD, (2) adult (>18 years old) study population, (3) ≥ 6-month follow-up, (4) paroxysmal AF, (5) preserved left ventricular ejection fraction (LVEF) and (6) reported 1 or more clinical outcomes. Observational studies, unpublished data, conference papers, case reports, editorials, reviews, expert opinions, and non-English studies were excluded.

### 2.3 Data extraction and quality appraisal

Two investigators (A.P and G.V) extracted data from each study using standardized protocol and reporting forms. Two reviewers (A.P and G.V) independently assessed the quality items, and disagreements were resolved by consensus. The quality of individual studies was assessed by two investigators (A.P and G.V) using the Cochrane Risk of Bias Tool version 2.0.

### 2.4 Study endpoints

The study endpoints were:

Atrial tachyarrhythmia (AT) recurrence, defined as any recurrent atrial arrhythmias (AF, atrial flutter or atrial tachycardia) lasting longer than 30 s at follow up after the initial 2–3 months blanking period post-ablation [20].

Progression to persistent AF was defined as the first AT occurrence lasting 7 days or longer or lasting 48 h to 7 days but necessitating cardioversion for termination.

Overall complications included stroke/transient ischemic attack (TIA), pericardial effusion/tamponade, phrenic-nerve palsy, syncope, wide-complex tachycardia or proarrhythmic event, pacemaker implantation due to sick sinus syndrome or atrioventricular block, vascular complications and clinically significant bleedings.

HF hospitalization was defined as HF relapse-related admission excluding hospitalization for AT recurrence. All-cause mortality was defined as death resulting from cardiovascular and other causes.

### 2.5 Statistical analysis

Descriptive statistics are presented as means and standard deviations (SD) for the continuous variables or a number of cases (n) and percentages (%) for the dichotomous and categorical variables. The Mantel–Haenszel Risk Ratio (RR) model was used to summarize the data for binary outcomes among the treatment arms. Summary estimates and 95 % confidence intervals (CI) were reported for the continuous variables as the standardized mean difference. The heterogeneity across studies was evaluated byusing the Chi^2^, Tau^2^, and Higgins-I^2^ statistics and random effects models of DerSimonian and Laird was used. Subgroup analyses were performed to assess potential sources of heterogeneity according to ablation energy [Cryoablation (Cryo) and Radiofrequency ablation (RF)] and first-line treatment with CA.

Publication bias was assessed by graphical inspection of funnel plots. The statistical analysis was performed using Review Manager (RevMan) (computer program) Version 5.4.1, Copenhagen, Denmark: Nordic Cochrane Centre, the Cochrane Collaboration, 2020.

## 3 Results

### 3.1 Study selection and Baseline Characteristics

**Fig. 1:**
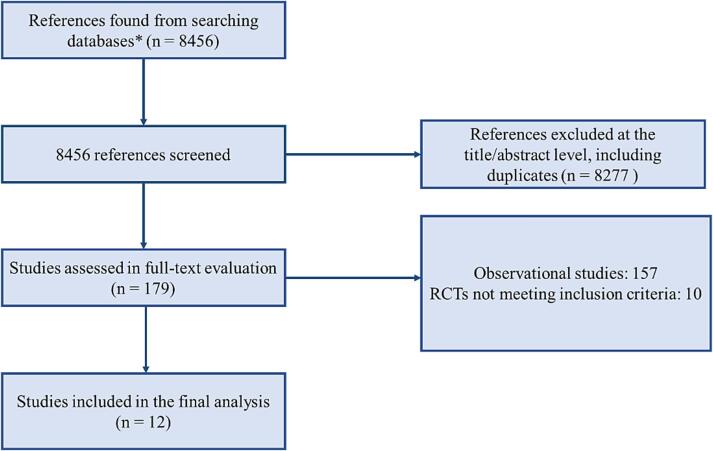
Evidence search and selection of the preferred reporting items for systematic reviews and *meta*-analyses (PRISMA). ***RCT:****randomized control trial.* * Medline, Embase, Scopus.

Among screened articles, full texts were retrieved and reviewed for possible inclusion; a total of 12 randomized trials [6], [7], [8], [9], [10], [11], [12], [13], [14], [15], [16], [17] fulfilled the selection criteria and were included in the final analysis (Fig. 1).

The studies enrolled 2393 patients (Group CA: 1253 patients; Group AAD: 1140 patients). Overall, 66.2 % (95 % CI: 62.7 – 70.2 %) patients were male with an average age of 60.1 years (95 % CI: 58.3–––62.5); mean LVEF was 60.2 % (95 % CI 58.5–62.0 %) and all patients had paroxysmal AF. The average follow-up time was of 30.5 months (95 % CI: 24.1–36.7). Further details on baseline characteristics of the studies population are reported in Table 1.

**Table 1:** Study Baseline Characteristics of Patients Included in the Analysis.

**First author**	**Trial**	**CA tecnology**	**First line CA**	**Patients (n)**		**Age (years), mean ± SD**		**LVEF (%), mean ± SD**		**Monitoring**	**Follow-up (m)**	**Outcomes**	**Source of fundings**
				**CA**	**AAD**	**CA**	**AAD**	**CA**	**AAD**				
**Andrade et al. 2021**	EARLY-AF	Cryo-ablation	Yes	154	149	57.7 ± 12.3	59.5 ± 10.6	59.6 ± 7.0	59.8 ± 7.6	Implantable loop recorder	36	Progression to persistent AF, recurrence of AT, AF burden, quality of life, health care utilization and serious adverse events	Cardiac Arrhythmia Network of Canada and others
**Ding et al. 2022**		Cryo-ablation	Yes	102	102	60.90 ± 7.89	60.74 ± 10.16	60.91 ± 4.71	59.96 ± 5.00	Periodical scheduled visits with 12-lead ECGs, 24-h holter and *trans*-telephonic monitoring	36	Progression to persistent AF, recurrence of AT and serious adverse events	Tianjin Key Medical Discipline (Specialty) Construction Project
**Kanagaratnam et al. 2023**		Cryo-ablation	No	108	103	59.7 ± 12.22	60.5 ± 10.34	58.3 ± 5.00	57.9 ± 5.60	Periodical scheduled visits and *trans*-telephonic monitoring	12	Any hospital episode related to treatment for AT, recurrence of AT and serious adverse events	British Heart Foundation Project Grant and Medtronic
**Kuck et al. 2021**	ATTEST	RF-ablation	No	128	127	67.8 ± 4.8	67.6 ± 4.6	61.8 ± 5.8	62.3 ± 5.2	Periodical scheduled visits with 12-lead ECGs and *trans*-telephonic monitoring	36	Progression to persistent AF, recurrence of AT and serious adverse events	Biosense Webster
**Kuniss et al. 2021**	Cryo-FIRST	Cryo-ablation	Yes	107	111	50.5 ± 13.1	54.1 ± 13.4	62.8 ± 5.4	63.7 ± 5.4	Periodical scheduled visits with 12-lead ECGs, 7-days Holter	12	Recurrence of AT and serious adverse events	Medtronic
**Morillo et al. 2014**	RAAFT-2	RF-ablation	Yes	66	61	56.3 ± 9.3	54.3 ± 11.7	61.4 ± 4.8	60.8 ± 7.0	Transtelephonic monitor system	24	Recurrence of AT, quality of life and serious adverse events	Biosense Webster and Population Health ResearchInstitute at McMaster University
**Nielsen et al. 2012**	MANTRA-PAF	RF-ablation	Yes	146	148	56 ± 9	54 ± 10	NA	NA	Periodical scheduled visits with 7-day Holter-monitor	24	Burden of AF, recurrence of AT, quality of life and serious adverse events	Danish Heart Foundation, Biosense Webster and Finnish Foundation for Cardiovascular Research
**Pappone et al. 2006**	APAF	RF-ablation	No	99	99	55 ± 10	57 ± 10	60 ± 8	61 ± 6	Periodical scheduled visits with 12-lead ECG and 48-hour Holter monitoring, and portable event monitor 12-lead ECG	48	Recurrence of AT, progression to persistent AF and serious adverse events	Arrhythmology Department, San Raffaele University Hospital.
**Sohara et al. 2016**		RF balloon-ablation	No	100	43	58.8 ± 10.4	61.0 ± 10.0	66.7 ± 6.1	66.5 ± 6.5	Periodical scheduled visits with 12-lead ECGs, 24-h Holter and portable electrocardiogram monitor	12	Recurrence of AT and serious adverse events	Toray Industries
**Wazni et al. 2020**	STOP-AF	Cryo-ablation	Yes	104	99	60.4 ± 11.2	61.6 ± 11.2	60.9 ± 6.0	61.1 ± 5.9	Periodical scheduled visits with 12-lead ECGs, 7-days Holter	12	Recurrence of AT, quality of life, health care utilization and serious adverse events	Medtronic
**Wazni et al. 2005**	RAAFT-1	RF-ablation	Yes	33	37	53 ± 8	54 ± 8	53 ± 5	54 ± 6	Periodical scheduled visits with 12-lead ECGs, 24-h Holter, *trans*-telephonic monitoring and portable electrocardiogram monitor	12	Recurrence of AT, hospitalization rate, quality of life and serious adverse events	Acuson
**Wilber et al. 2010**		RF-ablation	No	106	61	55.5 (53.7–57.3)	56.1 (52.9–59.4)	62.3 (60.4–64.3)	62.7 (60.7–64.7)	Periodical scheduled visits with 12-lead ECGs, Holter and *trans*-telephonic monitoring	12	Recurrence of AT and serious adverse events	Biosense Webster

***AAD:****antiarrhythmic drugs;****AF:****atrial fibrillation;****AT:****atrial tachyarrhythmia;****CA:****catheter ablation;****NA:****not available;****LVEF:****left ventricular ejection fraction;****mo:****months;****RF:****radiofrequency*.

### 3.2 Atrial tachyarrhythmia recurrence

**Fig. 2:**
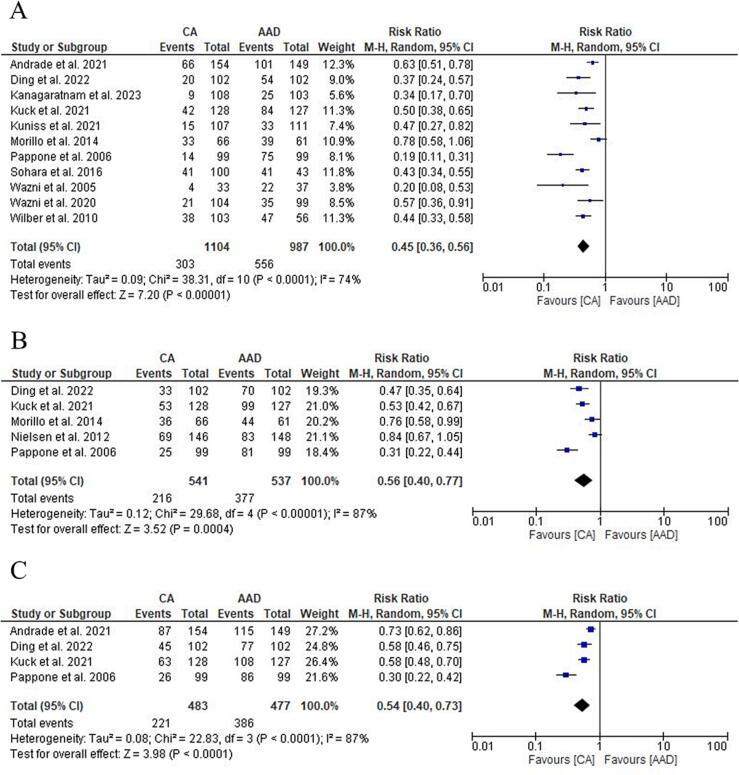
Forest plot comparing AT recurrence at 1 year (A), 2 years (B) and 3 years (C) between CA and AAD. ***AAD:****antiarrhythmic drugs;****AT:****atrial tachyarrhythmia;****CA:****catheter ablation.*

All RCTs reported data on AT recurrence. CA showed a significant reduction in AT recurrence compared to AAD at one year [27.4 % vs 56.3 %; RR: 0.45 (95 % CI: 0.46–0.56); p < 0.00001; I^2^ = 74 %], at two years [39.9 % vs 62.7 %; RR: 0.56 (95 % CI: 0.40–0.77); p = 0.0004; I^2^ = 87 %] and at three years [45.7 % vs 80.9 %; RR: 0.54 (95 % CI 0.40–0.73); p < 0.0001; I^2^ = 87 %] (Fig. 2 A-B-C). Subgroup analysis showed no significant difference between RF and Cryo at follow up (Supplemental Fig. 1 A-B-C). Considering RCTs with CA as first-line treatment, CA significantly reduced AT recurrences at 1 year [28 % vs 50.8 %; RR: 0.53 (95 % CI: 0.40–0.70); p = 0.0001; I^2^ = 66 %], two years [43.9 % vs 63.3 %; RR: 0.68 (95 % CI: 0.49–0.95); p = 0.02; I^2^ = 79 %] and three years [51.5 % vs 76.4 %; RR: 0.67 (95 % CI: 0.54–0.83); p = 0.0003; I^2^ = 56 %] compared to AAD (Supplemental Fig. 2 A-B-C). No significant reduction in heterogeneity was found in any subgroup analyses to assess potential sources of heterogeneity (Supplemental Figs. 1 and 2).

### 3.3 Progression to persistent AF

**Fig. 3:**
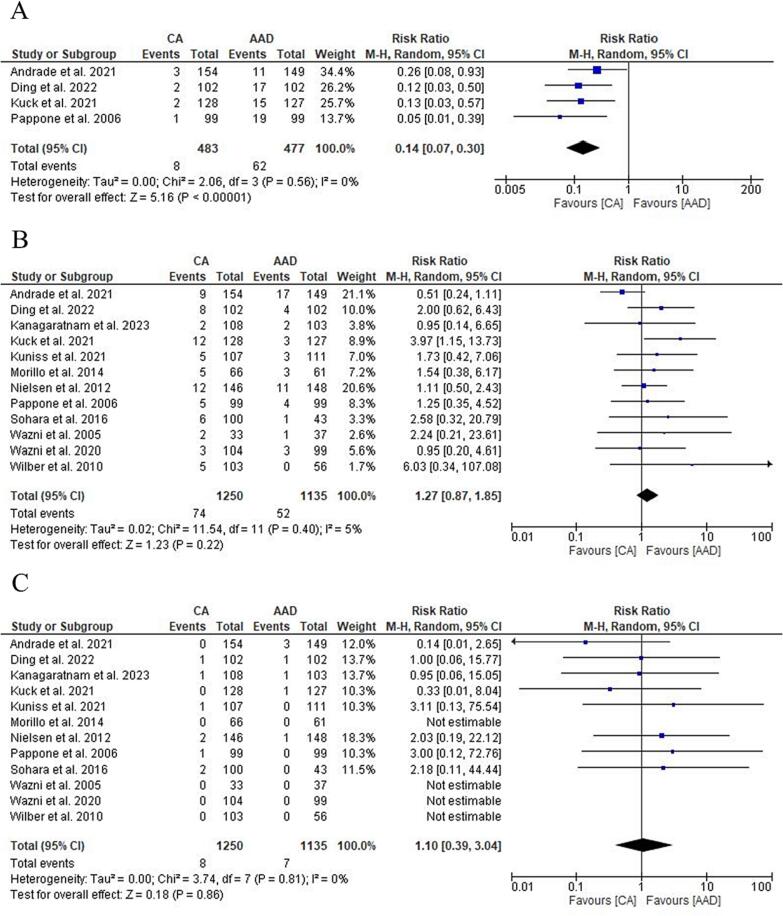
Forest plot comparing Progression to persistent Atrial Fibrillation (A), Overall Complications (B) and Stroke/TIA (C) between CA and AAD. ***AAD:****antiarrhythmic drugs;****AF:****Atrial Fibrillation;****CA:****catheter ablation.*

Four RCTs reported data on progression to persistent AF [6], [7], [9], [13]. CA significantly reduced the progression to persistent AF [1.6 % vs 12.9 %; RR: 0.14 (95 % CI: 0.07–0.30); p < 0.00001; I^2^ = 0 %] (Fig. 3 A). Subgroup analysis between RF and Cryo showed no significant difference (Supplemental Fig. 1 D). CA as a first-line treatment maintained a significant reduction in progression to persistent AF compared to AAD [1.9 % vs 11.1 %; RR: 0.19 (95 % CI: 0.07–0.48); p = 0.0005; I^2^ = 0 %] (Supplemental Fig. 2 D).

### 3.4 Overall complications

All RCTs reported data on overall complications. The most frequent adverse event in the CA group was pericardial effusion/tamponade (1.7 %) while in the AAD group was syncope (0.8 %). A summary of the overall complications is shown in Table 2. No differences were found in overall complication rate between CA and AAD [5.9 % vs 4.5 %; RR: 1.27 (95 % CI: 0.87–1.85); p = 0.22; I^2^ = 5 %] (Fig. 3 B) and in subgroup analysis (Supplemental Fig. 1 E, Supplemental Fig. 2 E).

**Table 2:** Complications.

**First author**	**Trial**	**Stroke/tia**		**Pericardial effusion/tamponation**		**Phrenic-nerve palsy**		**Sincope**		**Wide-complex tachycardia or proarrhythmic event**		**Bradycardia or arteriovenous block for which pacemaker insertion was warranted**		**Vascular event**		**Bleeding**	
		**CA**	**AAD**	**CA**	**AAD**	**CA**	**AAD**	**CA**	**AAD**	**CA**	**AAD**	**CA**	**AAD**	**CA**	**AAD**	**CA**	**AAD**
**Andrade et al. 2021**	EARLY-AF	0	3	0	1	3	0	1	3	0	2	2	4	2	0	0	1
**Ding et al. 2022**		1	1	1	0	2	0	0	0	0	0	0	0	3	0	1	3
**Kanagaratnam et al. 2023**		1	1	0	0	0	0	0	0	0	0	0	0	1	0	0	0
**Kuck et al. 2021**	ATTEST	0	1	6	0	0	0	0	0	0	0	0	0	4	0	1	2
**Kuniss et al. 2021**	Cryo-FIRST	1	0	3	0	0	1	0	1	0	1	0	0	1	0	0	0
**Morillo et al. 2014**	RAAFT-2	0	0	4	0	0	0	0	2	0	1	1	0	0	0	0	0
**Nielsen et al. 2012**	MANTRA-PAF	2	1	4	1	0	0	0	0	1	2	0	1	1	0	1	0
**Pappone et al. 2006**	APAF	1	0	1	0	0	0	0	0	0	0	0	0	3	0	0	0
**Sohara et al. 2016**		2	0	0	0	0	0	0	1	0	0	2	0	1	0	0	0
**Wazni et al. 2020**	STOP-AF	0	0	1	1	0	0	0	2	1	0	0	0	0	0	1	0
**Wazni et al. 2005**	RAAFT-1	0	0	0	0	0	0	0	0	0	0	0	0	0	0	2	1
**Wilber et al. 2010**		0	0	1	0	0	0	0	0	0	0	0	0	1	0	0	0

**Table 2.** Summary of overall complications in the included studies.

***AAD:****antiarrhythmic drugs;****CA:****catheter ablation*

### 3.5 Stroke/TIA

All RCTs reported data on stroke/TIA. No differences were found in Stroke/TIA rate between CA and AAD [0.6 % vs 0.6 %; RR: 1.10 (95 % CI: 0.39–3.04); p = 0.86; I^2^ = 0 %] (Fig. 3 C) with no differences in subgroup analysis too (Supplemental Fig. 1 F and 2F).

### 3.6 Bleedings

**Fig. 4:**
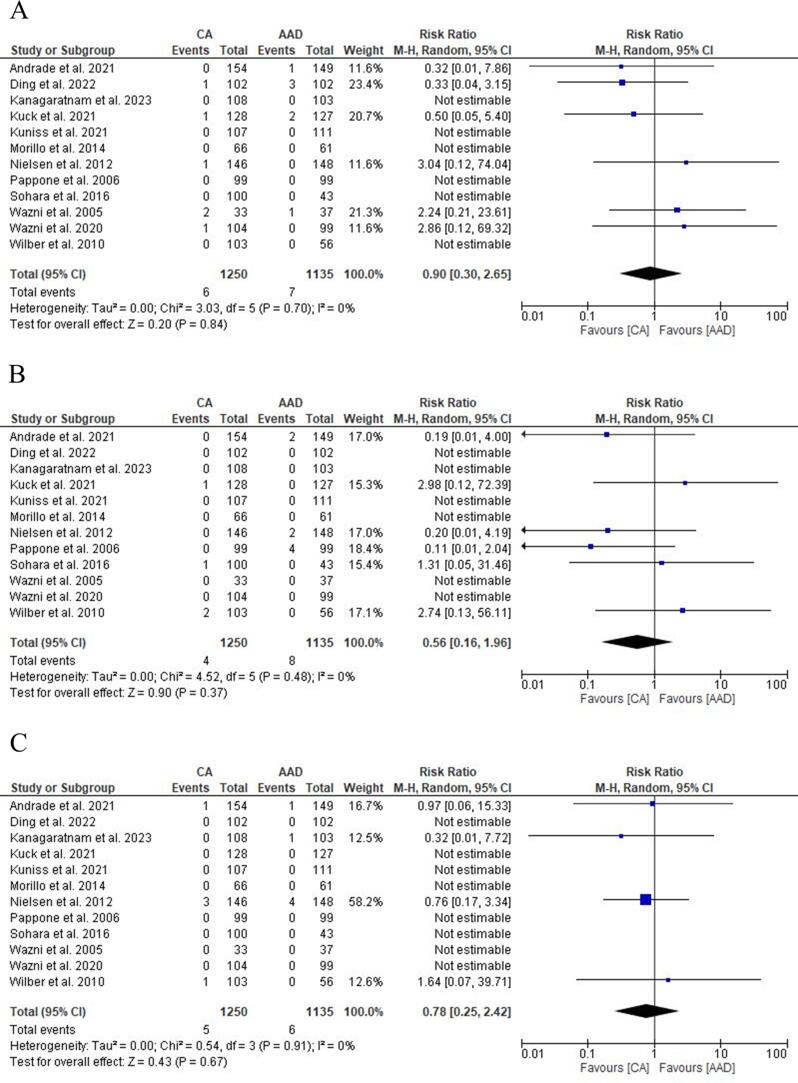
Forest plot comparing Bleedings (A), HF hospitalization (B), All-cause mortality (C) between CA and AAD. ***AAD:****antiarrhythmic drugs;****CA:****catheter ablation;****HF:****heart failure.*

All RCTs reported data on bleedings. No differences were found in bleedings rate between CA and AAD [0.4 % vs 0.6 %; RR: 0.90 (95 % CI: 0.30–2.65); p = 0.84; I^2^ = 0 %] (Fig. 4 A) with no differences in subgroup analysis too (Supplemental Fig. 1 G and 2 G).

### 3.7 HF hospitalization and all-cause mortality

All RCTs reported data on HF hospitalization and all-cause mortality. No difference was found in HF hospitalization [0.3 % vs 0.7 %; RR: 0.56 (95 % CI: 0.16–1.96); p = 0.37; I^2^ = 0 %] and all-cause mortality [0.4 % vs 0.5 %; RR: 0.78 (95 % CI: 0.25–2.42); p = 0.67; I^2^ = 0 %] between CA and ADD (Fig. 4 B-C). Subgroup analysis showed no significant differences in HF hospitalization or all-cause mortality (Supplemental Figs. 1-2 H-I).

### 3.8 Publication bias

**Fig. 5:**
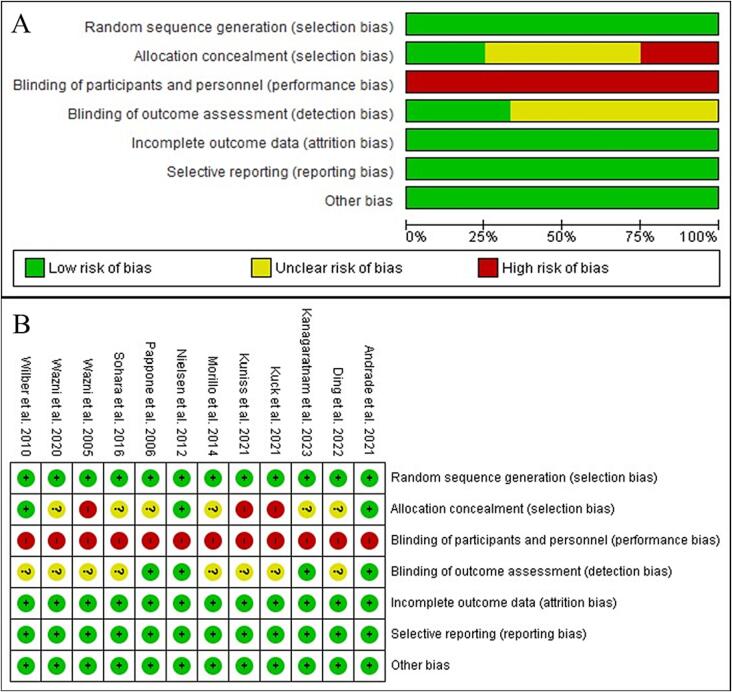
(A) Methodological quality graph and (B) methodological quality summary of the Cochrane Risk of Bias tool for Randomized Controlled Trials.

A graph and summary of Cochrane Risk of Bias tool for RCT is reported in Fig. 5. The funnel plots for visual inspection of the bias showed no bias (Supplemental Fig. 3).

## 4 Discussion

The aim of this updated *meta*-analysis was to evaluate the efficacy and safety of CA compared to AAD in the paroxysmal AF treatment in patients without SHD including only RCTs.

Specifically, CA showed to reduce AF recurrence rates at 1 year, 2 years, and 3 years, and the progression from paroxysmal to persistent AF with no difference in terms of safety and HF hospitalizations compared to AAD.

Furthermore, at the subgroup analysis, CA confirmed the superior efficacy regardless to the ablation energy employed, preserving a similar safety profile to AAD.

In addition, first-line CA of AF in our *meta*-analysis was confirmed as superior to AAD therapy in short- and long-term rhythm control, without resulting in reduced safety.

Our study, including 2393 patients, represents the *meta*-analysis with the largest number of RCTs comparing CA and AAD. In fact, previous recent *meta*-analyses included about half of the studies and patients and did not perform subgroup analyses by ablation energy and first-line approach [21], [22].

Our *meta*-analysis provides robust evidence supporting the superiority of CA over AAD therapy in terms of long-term AF recurrence rates across all time points evaluated. These findings highlight the long-term efficacy of CA in maintaining sinus rhythm and suggest higher efficacy in the management of paroxysmal AF compared to AAD.

Furthermore, our analysis revealed that CA significantly reduces the progression from paroxysmal to persistent AF. This is a notable finding, as the progression to persistent AF is associated with worse clinical outcomes and increased morbidity [5], [23]. Early AF ablation may alter the natural course of the disease, as pulmonary venous isolation, modulation of the autonomic nervous system and electro-anatomical substrate modification may favour a substantial reversal of adverse structural atrial remodelling [24]. Therefore, the ability of CA to prevent or delay this progression represents a significant advantage over AAD therapy leading to improved patient outcomes, avoiding AF ablation in the setting of persistent AF, characterized by less effectiveness than in paroxysmal AF [25].

Our *meta*-analysis did not find any significant differences in AF recurrence rates or complications when comparing RF and Cryo technologies for catheter ablation. This finding confirms that the choice of energy source does not significantly impact the efficacy or safety of the procedure, as already observed in the FIRE AND ICE Trial [26]. Nevertheless, evidence suggests that new technologies may be more efficient [27], [28]. In addition, the development of new non-thermal tissue-selective energies such as pulsed field ablation would provide excellent efficacy and safety [29].

Although no difference has been shown in terms of complications between CA and AAD, the *meta*-analytic cohort primarily consisted of relatively young individuals experiencing symptoms, without evident underlying SHD. For instance, the median age in the CABANA trial [30] differed significantly from the current study's population (67.5 years versus 60 years), with a 15 % in heart failure cases and 82 % of patients with CHA_2_DS_2_-VASc score ≥ 2. Nevertheless, a recent sub-analysis of EAST-AFNET 4 [31] showed that early rhythm control in patients with CHA_2_DS_2_-VASc score ≥ 4 reduced the primary composite efficacy outcome of cardiovascular death, stroke or hospitalisation for worsening heart failure or acute coronary syndrome, but not in patients with CHA_2_DS_2_-VASc score < 4. Furthermore, the primary safety outcome (death, stroke or serious adverse events of rhythm control therapy) was not different between study groups in patients with CHA_2_DS_2_-VASc score ≥ 4 but occurred more often in patients with CHA_2_DS_2_-VASc score < 4 randomised to early rhythm control. However, looking at the serious adverse events, these seem to be mainly due to AAD rather than CA (torsade de pointes, drug toxicity, drug-induced bradycardia, drug-induced atrioventricular block and syncope). These findings suggest that rhythm control therapy is associated with a better net clinical benefit in patients with multiple comorbidities than in patients with fewer comorbidities, indeed few events of HF hospitalisations and deaths occurred in our *meta*-analysis. Moreover, in terms of complications, AAD might have a comparable if not higher risk of adverse effects in patients with less comorbidity than with more comorbidities. However, as CA and AAD are associated with different types of complications, it is not possible to make a relevant comparison.

### 4.1 Limitations

It is important to consider certain limitations of our *meta*-analysis. None of the studies specified blinding of patients and it is possible that the post-ablation medical management differed between RCTs. Furthermore, some studies were open label and with unblinded outcome assessment. However, though patients and researchers were not subjected to blinding regarding treatment allocation and outcome, this was not considered sufficient to determine that these studies are at high risk of bias with regard to the outcomes of interest in this *meta*-analysis, which are relatively resistant to bias due to lack of blinding. Our *meta*-analysis reported high heterogeneity for AT recurrence at follow-up without reduction at subgroup analysis. In part, this could be due to the methodology used for assessing AT recurrences in the different studies (loop recorder, Holter ECG 24, periodic scheduled visits), which could potentially misestimate AT recurrence rates. Additional ablation outside the PVs, performed in some RCTs, could have affected the clinical outcomes [32]. The RCTs included here enrolled patients from 2006 to 2022, involving temporal changes in both CA and drug therapy.

## 5 Conclusions

In conclusion, our *meta*-analysis of RCTs provides compelling evidence supporting the superiority of CA over AAD therapy for the treatment of paroxysmal AF. CA demonstrated lower rates of AT recurrence at 1 year, 2 years, and 3 years, as well as a significant reduction in the progression from paroxysmal to persistent AF, with no difference for safety in comparison with AAD. Importantly, the choice between RF and Cryo technologies did not affect efficacy and safety, underlining that both technologies are equally effective and safe.

## Funding statement

Open Access Funding provided by “Università degli Studi di Messina” within the CRUI-CARE Agreement.

## Declaration of Competing Interest

The authors declare the following financial interests/personal relationships which may be considered as potential competing interests: Dr. Chierchia received compensation for teaching purposes and proctoringfrom Medtronic, Abbott, Biotronik, Boston Scientific, and Acutus Medical. Dr. de Asmundis receives research grants on behalf of the center from Biotronik, Medtronic, Abbott, LivaNova, BostonScientific, AtriCure, Philips, and Acutus, and compensation for teaching purposes and proctoring from Medtronic, Abbott, Biotronik, Livanova, Boston Scientific, Atricure, Acutus Medical, and Daiichi Sankyo.

## References

[1] Hindricks G., Potpara T., Dagres N., Arbelo E., Bax J.J., Blomström-Lundqvist C., Boriani G., Castella M., Dan G.-A., Dilaveris P.E. (2021). 2020 ESC Guidelines for the diagnosis and management of atrial fibrillation developed in collaboration with the European Association for Cardio-Thoracic Surgery (EACTS). Eur. Heart J..

[2] Magnocavallo M., Parlavecchio A., Vetta G., Gianni C., Polselli M., De Vuono F., Pannone L., Mohanty S., Cauti F.M., Caminiti R. (2022). Catheter Ablation versus Medical Therapy of Atrial Fibrillation in Patients with Heart Failure: An Updated Systematic Review and Meta-Analysis of Randomized Controlled Trials. JCM..

[3] Vetta G., Parlavecchio A., Caminiti R., Crea P., Magnocavallo M., Della Rocca D.G., Lavalle C., Vetta F., Marano G., Ruggieri C. (2022). Non-conducted premature atrial complexes: A new independent predictor of atrial fibrillation in cryptogenic stroke. J. Electrocardiol..

[4] Lucà F., Colivicchi F., Oliva F., Abrignani M., Caretta G., Di Fusco S.A., Giubilato S., Cornara S., Di Nora C., Pozzi A. (2023). Management of oral anticoagulant therapy after intracranial hemorrhage in patients with atrial fibrillation. Front. Cardiovasc. Med..

[5] Kirchhof P., Camm A.J., Goette A., Brandes A., Eckardt L., Elvan A., Fetsch T., van Gelder I.C., Haase D., Haegeli L.M. (2020). Early Rhythm-Control Therapy in Patients with Atrial Fibrillation. N. Engl. J. Med..

[6] Andrade J.G., Deyell M.W., Macle L., Wells G.A., Bennett M., Essebag V., Champagne J., Roux J.-F., Yung D., Skanes A. (2023). Progression of Atrial Fibrillation after Cryoablation or Drug Therapy. N. Engl. J. Med..

[7] Ding J., Cheng A., Li P., Yan Y., Shi Y., Xue Z., Sun S., Xu J. (2022). Cryoballoon catheter ablation or drug therapy to delay progression of atrial fibrillation: A single-center randomized trial. Front. Cardiovasc. Med..

[8] Kanagaratnam P., McCready J., Tayebjee M., Shepherd E., Sasikaran T., Todd D., Johnson N., Kyriacou A., Hayat S., Hobson N.A. (2023). Ablation versus anti-arrhythmic therapy for reducing all hospital episodes from recurrent atrial fibrillation: a prospective, randomized, multi-centre, open label trial. EP Europace..

[9] Kuck K.-H., Lebedev D.S., Mikhaylov E.N., Romanov A., Gellér L., Kalējs O., Neumann T., Davtyan K., On Y.K., Popov S. (2021). Catheter ablation or medical therapy to delay progression of atrial fibrillation: the randomized controlled atrial fibrillation progression trial (ATTEST). EP Europace..

[10] Kuniss M., Pavlovic N., Velagic V., Hermida J.S., Healey S., Arena G., Badenco N., Meyer C., Chen J., Iacopino S. (2021). Cryoballoon ablation vs. antiarrhythmic drugs: first-line therapy for patients with paroxysmal atrial fibrillation. EP Europace..

[11] Morillo C.A., Verma A., Connolly S.J., Kuck K.H., Nair G.M., Champagne J., Sterns L.D., Beresh H., Healey J.S., Natale A. (2014). Radiofrequency Ablation vs Antiarrhythmic Drugs as First-Line Treatment of Paroxysmal Atrial Fibrillation (RAAFT-2): A Randomized Trial. JAMA.

[12] Nielsen J.C., Kristensen L., Andersen H.R., Mortensen P.T., Pedersen O.L., Pedersen A.K. (2003). A randomized comparison of atrial and dual-chamber pacing in 177 consecutive patients with sick sinus syndrome: echocardiographic and clinical outcome. J. Am. Coll. Cardiol..

[13] Pappone C., Vicedomini G., Augello G., Manguso F., Saviano M., Baldi M., Petretta A., Giannelli L., Calovic Z., Guluta V. (2011). Radiofrequency Catheter Ablation and Antiarrhythmic Drug Therapy: A Prospective, Randomized, 4-Year Follow-Up Trial: The APAF Study. Circ: Arrhythmia Electrophysiol..

[14] Wazni O.M., Dandamudi G., Sood N., Hoyt R., Tyler J., Durrani S., Niebauer M., Makati K., Halperin B., Gauri A. (2021). Cryoballoon Ablation as Initial Therapy for Atrial Fibrillation. N. Engl. J. Med..

[15] Wazni O.M., Marrouche N.F., Martin D.O., Verma A., Bhargava M., Saliba W., Bash D., Schweikert R., Brachmann J., Gunther J. (2005). Radiofrequency ablation vs antiarrhythmic drugs as first-line treatment of symptomatic atrial fibrillation: a randomized trial. JAMA.

[16] Wilber D.J., Pappone C., Neuzil P., De Paola A., Marchlinski F., Natale A., Macle L., Daoud E.G., Calkins H., Hall B. (2010). Comparison of Antiarrhythmic Drug Therapy and Radiofrequency Catheter Ablation in Patients With Paroxysmal Atrial Fibrillation: A Randomized Controlled Trial. JAMA.

[17] Sohara H., Ohe T., Okumura K., Naito S., Hirao K., Shoda M., Kobayashi Y., Yamauchi Y., Yamaguchi Y., Kuwahara T. (2016). HotBalloon Ablation of the Pulmonary Veins for Paroxysmal AF. J. Am. Coll. Cardiol..

[18] Cosedis Nielsen J., Johannessen A., Raatikainen P., Hindricks G., Walfridsson H., Kongstad O., Pehrson S., Englund A., Hartikainen J., Mortensen L.S. (2012). Radiofrequency Ablation as Initial Therapy in Paroxysmal Atrial Fibrillation. N. Engl. J. Med..

[19] M.J. Page, J.E. McKenzie, P.M. Bossuyt, I. Boutron, T.C. Hoffmann, C.D. Mulrow, L. Shamseer, J.M. Tetzlaff, E.A. Akl, S.E. Brennan, et al. The PRISMA 2020 statement: an updated guideline for reporting systematic reviews, BMJ 2021 (2020) n71. 10.1136/bmj.n71 PMC8005924 33782057

[20] Bordignon S., Barra S., Providencia R., de Asmundis C., Marijon E., Farkowski M.M., Anic A., Guerra J.M., Kosiuk J., Iliodromitis K. (2022). The blanking period after atrial fibrillation ablation: an European Heart Rhythm Association survey on contemporary definition and management. EP Europace..

[21] Turagam M.K., Musikantow D., Whang W., Koruth J.S., Miller M.A., Langan M.-N., Sofi A., Choudry S., Dukkipati S.R., Reddy V.Y. (2021). Assessment of Catheter Ablation or Antiarrhythmic Drugs for First-line Therapy of Atrial Fibrillation: A Meta-analysis of Randomized Clinical Trials. JAMACardiol..

[22] Kheiri B., Simpson T.F., Przybylowicz R., Merrill M., Alhamoud H., Osman M., Dalouk K., Stecker E., Henrikson C.A., Nazer B. (2021). Ablation Versus Antiarrhythmic Drugs as First-Line Treatment of Paroxysmal Atrial Fibrillation: A Meta-Analysis of Randomized Trials. Circ. Arrhythm. Electrophysiol..

[23] Gunawardene M.A., Willems S. (2022). Atrial fibrillation progression and the importance of early treatment for improving clinical outcomes. EP Europace..

[24] Walters T.E., Nisbet A., Morris G.M., Tan G., Mearns M., Teo E., Lewis N., Ng A., Gould P., Lee G. (2016). Progression of atrial remodeling in patients with high-burden atrial fibrillation: Implications for early ablative intervention. Heart Rhythm..

[25] Verma A., Jiang C., Betts T.R., Chen J., Deisenhofer I., Mantovan R., Macle L., Morillo C.A., Haverkamp W., Weerasooriya R. (2015). Approaches to Catheter Ablation for Persistent Atrial Fibrillation. N. Engl. J. Med..

[26] Kuck K.-H., Brugada J., Fürnkranz A., Metzner A., Ouyang F., Chun K.R.J., Elvan A., Arentz T., Bestehorn K., Pocock S.J. (2016). Cryoballoon or Radiofrequency Ablation for Paroxysmal Atrial Fibrillation. N. Engl. J. Med..

[27] Lee A.C., Voskoboinik A., Cheung C.C., Yogi S., Tseng Z.H., Moss J.D., Dewland T.A., Lee B.K., Lee R.J., Hsia H.H. (2023).

[28] Parlavecchio A., Vetta G., Coluccia G., Pistelli L., Caminiti R., Ajello M., Magnocavallo M., Dattilo G., Foti R., Carerj S. (2023). High power short duration versus low power long duration ablation in patients with atrial fibrillation: A meta-analysis of randomized trials. Pacing Clin. Electrophysiol..

[29] Reddy V.Y., Gerstenfeld E.P., Natale A., Whang W., Cuoco F.A., Patel C., Mountantonakis S.E., Gibson D.N., Harding J.D., Ellis C.R. (2023). Pulsed Field or Conventional Thermal Ablation for Paroxysmal Atrial Fibrillation. N Engl J Med..

[30] Packer D.L., Mark D.B., Robb R.A., Monahan K.H., Bahnson T.D., Poole J.E., Noseworthy P.A., Rosenberg Y.D., Jeffries N., Mitchell L.B. (2019). Effect of Catheter Ablation vs Antiarrhythmic Drug Therapy on Mortality, Stroke, Bleeding, and Cardiac Arrest Among Patients With Atrial Fibrillation: The CABANA Randomized Clinical Trial. JAMA.

[31] Rillig A., Borof K., Breithardt G., Camm A.J., Crijns H.J.G.M., Goette A., Kuck K.-H., Metzner A., Vardas P., Vettorazzi E. (2022). Early Rhythm Control in Patients With Atrial Fibrillation and High Comorbidity Burden. Circulation.

[32] Della Rocca D.G., Di Biase L., Mohanty S., Trivedi C., Gianni C., Romero J., Tarantino N., Magnocavallo M., Bassiouny M., Natale V.N. (2021). Targeting non-pulmonary vein triggers in persistent atrial fibrillation: results from a prospective, multicentre, observational registry. EP Europace..

