# Supplementary material for: Catheter ablation in patients with paroxysmal atrial fibrillation and absence of structural heart disease: A meta-analysis of randomized trials
Source: Int J Cardiol Heart Vasc. 2023 Nov 5;49:101292. doi: 10.1016/j.ijcha.2023.101292 (PMC10656266; doi:10.1016/j.ijcha.2023.101292)
Supplement: Supplementary data 1 [file mmc1.docx]

**SUPPLEMENTAL MATERIALS**

**Catheter Ablation in Patients with Paroxysmal Atrial Fibrillation: A Meta-Analysis of Randomized Trials**

**Expanded Methods**

- *Data Sources and Searches*

**Supplemental Figures**

- *Supplemental Figure 1*.
- *Supplemental Figure 2.*
- *Supplemental Figure 3.*
- *Supplemental Figure 4.*

**Expanded Methods**

*Data Sources and Searches*

We used the following search strings respectively for Pubmed (1), Embase (2), Scopus (3):

1) (((((Paroxysmal Atrial Fibrillation) AND (Catheter ablation)) OR (antiarrhythmic drugs)) OR (Radiofrequency ablation)) OR (RF) OR (Atrial Fibrillation Ablation))))

2) (((Mesh descriptor: [Paroxysmal Atrial Fibrillation] explode all trees) OR ((Catheter ablation) :ti OR (Catheter ablation and antiarrhythmic drugs n):ti OR (Radiofrequency ablation) OR (antiarrhythmic drugs)))

3) (Catheter ablation or antiarrhythmic drugs).ti. and (RF and Pulmonary Vein Isolation or PVI or Atrial Fibrillation Ablation).ab.

***Supplemental Figure 1.*** Forest plot comparing AT recurrence at 1 year (A), 2 years (B), 3 years (C), Progression to persistent AF (D), Overall Complications (E), Stroke/TIA (F), Bleedings (G), HF hospitalization (H) and All-cause mortality (I) according to ablation energy.


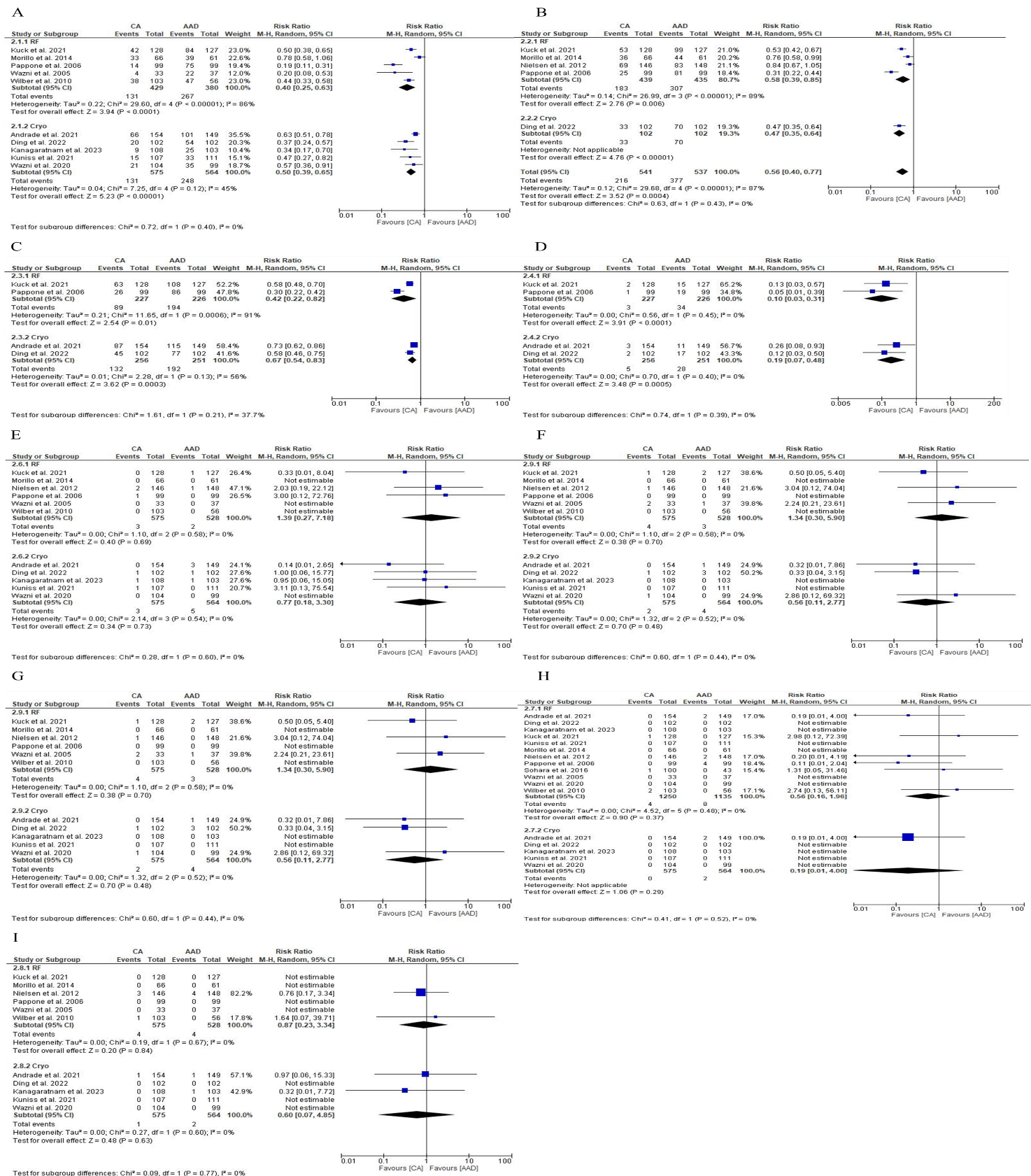


***AAD:*** *antiarrhythmic drugs;* ***AF:*** *atrial Fibrillation;* ***AT:*** *atrial tachyarrhythmia;* ***CA:*** *catheter ablation;* ***HF:*** *heart failure.*

***Supplemental Figure 2.*** Forest plot comparing AT recurrence at 1 year (A), 2 years (B), 3 years (C), Progression to persistent AF (D), Overall Complications (E), Stroke/TIA (F), Bleedings (G), HF hospitalization (H), All-cause mortality (I) between first-pass CA and AAD.


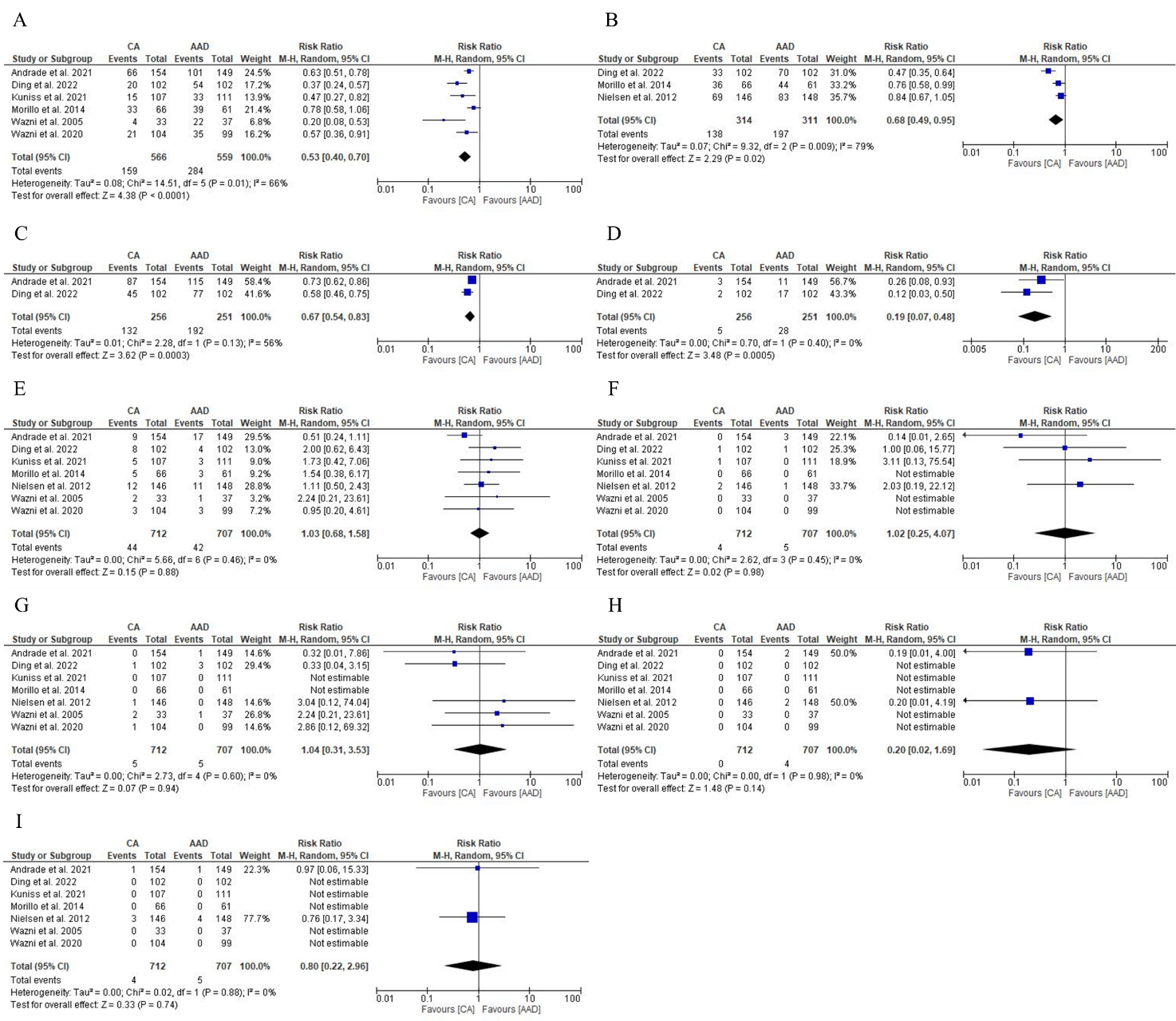
 ***AAD:*** *antiarrhythmic drugs;* ***AF:*** *atrial Fibrillation;* ***AT:*** *atrial tachyarrhythmia;* ***CA:*** *catheter ablation;* ***HF:*** *heart failure.*

***Supplemental Figure 3*.** Funnel plots for visual inspection of the bias regarding AT recurrence (A), Progression to persistent AF (B), Overall Complications (C), Stroke/TIA (D), Bleedings (E), HF hospitalization (F), All-cause mortality (G) between CA and AAD.


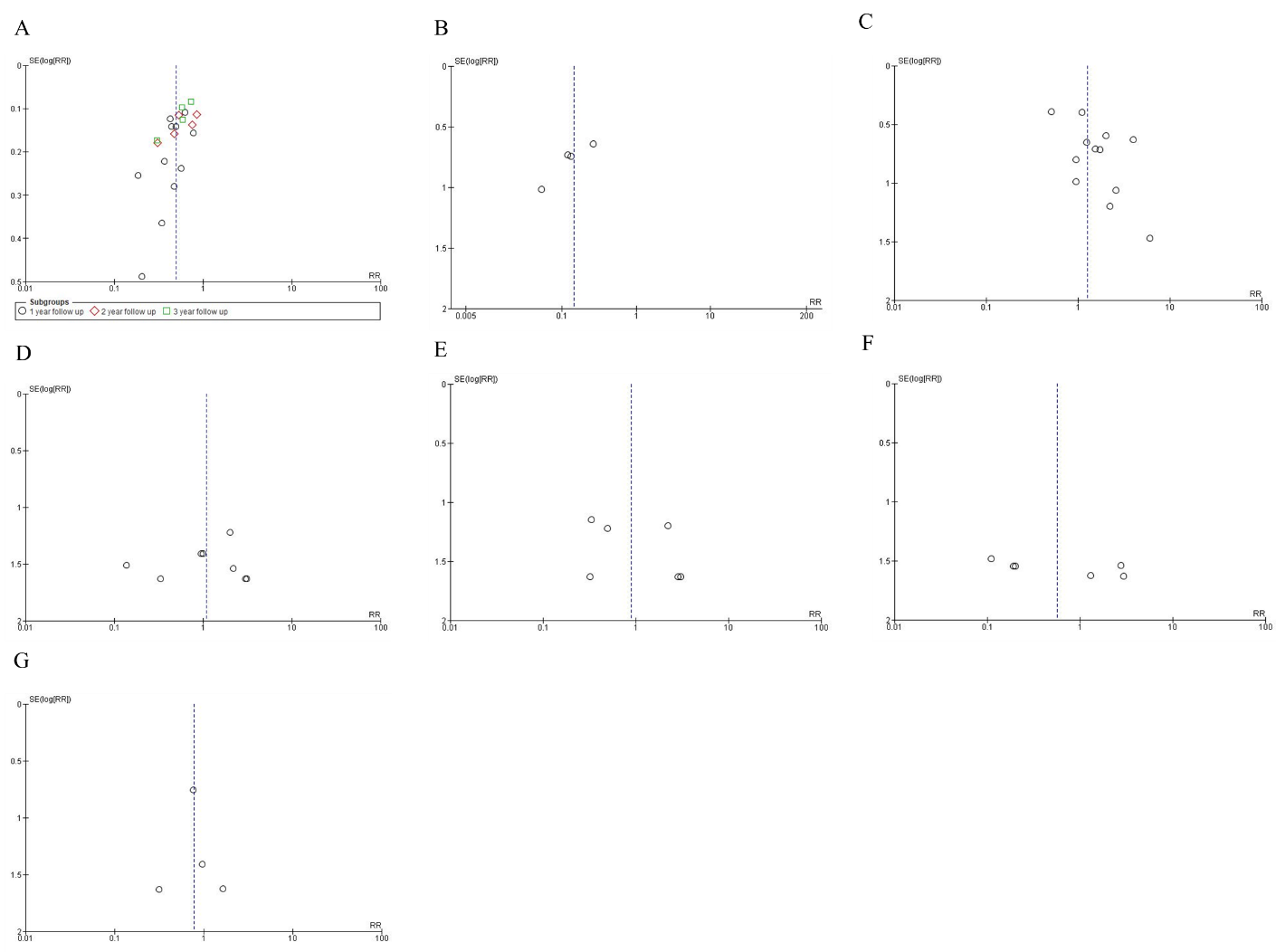


***AAD:*** *antiarrhythmic drugs;* ***AF:*** *atrial Fibrillation;* ***AT:*** *atrial tachyarrhythmia;* ***CA:*** *catheter ablation;* ***HF:*** *heart failure.*
